# Anticholinergic burden quantified using the Japanese risk scale as a predictor of frailty and sarcopenia among community‐dwelling older adults: A 9‐year Kashiwa cohort study

**DOI:** 10.1111/ggi.70012

**Published:** 2025-03-06

**Authors:** Tomoki Tanaka, Masahiro Akishita, Taro Kojima, Bo‐Kyung Son, Katsuya Iijima

**Affiliations:** ^1^ Institute of Gerontology The University of Tokyo Tokyo Japan; ^2^ Department of Geriatric Medicine Tokyo Metropolitan Geriatric Hospital and Institute of Gerontology Tokyo Japan; ^3^ Department of Geriatric Medicine, Graduate School of Medicine The University of Tokyo Tokyo Japan; ^4^ Department of Geriatric Medicine, School of Medicine International University of Health and Welfare Narita Japan; ^5^ Institute for Future Initiatives The University of Tokyo Tokyo Japan

**Keywords:** anticholinergic burden, frailty, polypharmacy, potentially inappropriate medication, sarcopenia

## Abstract

**Aim:**

Given the adverse effects of anticholinergic drugs and the necessity for medication evaluation tools in the aging population, a comprehensive scale to assess the total anticholinergic burden in Japan was developed. We examined the longitudinal association between the anticholinergic burden, quantified using the Japanese Anticholinergic Drug Risk Scale, and the development of frailty and sarcopenia in older adults.

**Methods:**

In this longitudinal population‐based cohort study, 2044 older residents without long‐term care needs were randomly selected from a community in Kashiwa, Japan. Baseline data were collected in 2012, with follow‐ups in 2013, 2014, 2016, 2018, and 2021. Medications were identified through interviews and assessed with the Screening Tool for Older Persons' Appropriate Prescriptions for the Japanese. The anticholinergic burden was quantified using the Japanese Anticholinergic Risk Scale. We evaluated new‐onset frailty and sarcopenia using the Cardiovascular Health Study Index and Asian Working Group for Sarcopenia 2019 criteria, respectively.

**Results:**

Of the 1549 participants without sarcopenia or frailty at baseline (age 72.5 ± 5.5 years; 49.1% women; median follow‐up 6.0 years), 274 and 230 developed new‐onset frailty and sarcopenia, respectively, during follow‐up. After adjusting for potential confounders, an anticholinergic burden score ≥3 was strongly associated with new‐onset frailty and sarcopenia (adjusted hazard ratio [95% confidence interval]: 2.45 [1.52–3.94] and 2.01 [1.20–3.35], respectively).

**Conclusions:**

Anticholinergic burden is a predictor of frailty and sarcopenia in community‐dwelling older adults. Effective evaluation and management of anticholinergic burden using the Japanese Anticholinergic Drug Risk Scale are crucial for promoting healthy aging and mitigating adverse health outcomes. **Geriatr Gerontol Int 2025; 25: 520–527**.

## Graphical abstract

Anticholinergic burden, quantified using the Japanese Anticholinergic Drug Risk Scale, may be a predictor of frailty and sarcopenia in community‐dwelling older adults. Effective evaluation and management of anticholinergic burden using the Japanese Anticholinergic Drug Risk Scale are crucial for promoting healthy aging and mitigating adverse health outcomes.

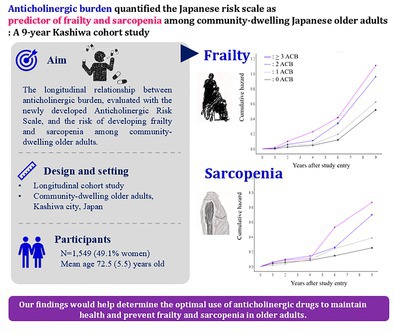

## Introduction

In an aging society, the functional capacity of older adults is a critical concern. Frailty, indicating a decline in physical strength, mental capacity, and social support, includes sarcopenia as a key component.^1^, ^2^ Sarcopenia is characterized by age‐related declines in skeletal muscle mass and strength; it also significantly impacts the independence and quality of life of older adults.^2^, ^3^ Both conditions heighten the likelihood of a requirement for long‐term care,^4^, ^5^ thereby increasing medical and caregiving costs, which constitute societal challenges.^6^

Moreover, the increase in polypharmacy in the aging population is primarily attributable to the prevalence of multiple comorbidities. Adverse drug events, which are typically preventable, are increasingly prevalent among older adults in outpatient settings;^7^ therefore, interventions by healthcare facilities, local governments, and national policies are warranted. Among these, anticholinergic drugs, commonly prescribed for various diseases, often go unnoticed but pose significant risks. These drugs inhibit acetylcholine and impact the autonomic nervous system, thereby resulting in conditions such as cognitive decline, urinary incontinence, and dry mouth.^8^ Excessive anticholinergic drug use can lead to severe outcomes, including impaired mobility, dementia, and increased mortality in older adults.^9^, ^10^, ^11^, ^12^, ^13^ Furthermore, polypharmacy and the use of potentially inappropriate medications have been identified as risk factors for new‐onset sarcopenia and frailty,^14^, ^15^ further emphasizing the need for a comprehensive scale to evaluate the total anticholinergic burden.

Consequently, The Japan Geriatrics Society has updated its guidelines to reflect current medical and medication scenarios, including the 2015 revision of the Screening Tool for Older Persons' Appropriate Prescriptions for the Japanese (STOPP‐J).^16^ Concurrently, international scales have been developed to assess the anticholinergic effects of medications and calculate total anticholinergic load. Responding to these developments, the Japanese Society of Geriatric Pharmacology introduced a tailored Anticholinergic Risk Scale in 2023, designed for easy evaluation by community healthcare and pharmacy professionals to promote appropriate anticholinergic drug usage. Nevertheless, a comprehensive analysis of the association between anticholinergic drug use^8^ and the combined outcomes of frailty and sarcopenia in aging populations is lacking.

Therefore, we conducted this study to explore the longitudinal relationship between anticholinergic burden, evaluated using the newly developed Anticholinergic Risk Scale, and the risk of developing frailty and sarcopenia among community‐dwelling older adults. We believe our findings could help determine the optimal use of anticholinergic drugs to maintain health and prevent frailty and sarcopenia in older adults.

## Methods

### Study setting and participants

Data from a longitudinal prospective cohort study conducted in Kashiwa, Japan, were employed to identify factors affecting healthy aging in community‐dwelling older adults.^17^ The area comprises both urban and rural communities. In 2012, 12 000 adults aged ≥65 with no long‐term care needs were randomly selected from the registry of Kashiwa, Japan, and invited to participate in the study via mail. A total of 2044 older adults (1013 men and 1031 women) consented to participate. The age distribution of the participants matched that of the general population in Kashiwa for each sex. Baseline data were collected at welfare and community centers between September and November 2012. Exclusion criteria included cognitive impairment (Mini‐Mental State Examination [MMSE] score <18), presence of frailty or sarcopenia at baseline, an implanted pacemaker (prohibiting bioelectrical impedance analysis for sarcopenia diagnosis), missing any follow‐up studies, and missing data on outcomes or medications. The participants were followed up in 2013, 2014, 2016, 2018, and 2021 to examine the longitudinal association between prescribed medications and outcomes. The study adhered to the Strengthening the Reporting of Observational Studies in Epidemiology (STROBE) reporting guidelines for cohort studies.

The study protocol was approved by the Ethics Committee of the University of Tokyo Life Science Research Center (21‐192).

### Prescribed medications and chronic diseases

Prescribed medication names and numbers were determined via personal interviews conducted by trained nurses using a standardized questionnaire. Participants maintaining prescription records were requested to present these documents at the time of assessment. In this study, we defined ≥5 drug prescriptions as polypharmacy. Medication counts were reassessed at each subsequent follow‐up, and participants were excluded from the polypharmacy category when prescription numbers were significantly reduced. Potentially inappropriate medications (PIMs) were defined as STOPP‐J‐listed drugs.^16^ The investigated medications were classified as “PIMs” or “not PIMs.” Total anticholinergic burden (ACB) was assessed using the Japanese version of the Anticholinergic Drug Risk Scale.^8^ This scale was developed based on 16 international scales, evaluating 286 drugs, and the scoring system was determined by averaging scores across the scales and the Delphi method.^8^ In this scale, 158 drugs used in Japan are assigned scores (37 drugs with a score of 3; 27 drugs with a score of 2; and 94 drugs with a score of 1). This Japanese scale covers only oral and patch medications with systemic action.^8^ Given that older adults often have multiple diseases and are prescribed multiple medications, the scores for each medication were summed to calculate the total ACB of the patient. Data on current chronic diseases (hypertension, diabetes mellitus, dyslipidemia, osteoporosis, malignant neoplasm, stroke, chronic renal failure, and heart disease) were also obtained during interviews, with comorbidity defined as the presence of two or more of these conditions.

### Outcomes

Frailty was assessed according to the Cardiovascular Health Study Index criteria, which define physical frailty based on five conditions: shrinking, exhaustion, low activity, weakness, and slowness.^1^ Participants without any of these conditions were classified as non‐frail, those with one or two conditions were classified as pre‐frail, and those with three or more conditions were classified as physically frail. This five‐item assessment method has been previously described.^17^ Frailty was evaluated at each follow‐up survey to identify new‐onset cases. The onset of frailty was recorded as the number of years from the start of the follow‐up period.

Sarcopenia was diagnosed according to the Asian criteria and cutoff threshold (Asian Working Group of Sarcopenia 2019);^3^ sarcopenia was identified as a low appendicular skeletal muscle mass with concomitant low muscle strength or physical function. Bioimpedance analyses were performed to assess low appendicular skeletal muscle mass, defined as <7.0 kg/m^2^ for men and <5.7 kg/m^2^ for women, using the InBody 420 body composition analyzer (InBody Japan, Tokyo, Japan). Low muscle strength (<28 kg for men and <18 kg for women) was measured using a Smedley‐type grip strength meter (Grip D dynamometer; Takei Scientific Instruments Co., Ltd, Niigata, Japan). Low physical function, defined as an average walking speed of <1.0 m/s, was determined based on the time required to travel 5 m between 11m lane. The time of the first development of sarcopenia was defined as the number of years of follow‐up.

### Covariates

The following covariates were included to control for their potential influence on the primary outcomes: age (years), sex, body mass index (BMI; kg/m^2^), educational level (categorized as having a college degree or lower), living arrangement (alone or with others), annual income (either ≥ or <1.4 million yen per household for men, and 1.2 million yen for women), cognitive function determined using MMSE scores,^18^ depressive symptoms assessed using the Geriatric Depression Scale‐15 (GDS‐15),^19^ exercising habit (engaging in physical activity at least once weekly during leisure time) evaluated using the Global Physical Activity Questionnaire,^20^ daily food diversity, and current alcohol consumption habits (answering “yes” to the question “Do you drink alcohol?”). Additionally, biochemical parameters such as serum albumin, total cholesterol, hemoglobin, C‐reactive protein, platelet count, fasting blood glucose, and systolic/diastolic blood pressure were collected through medical interviews and blood tests.

### Statistical analysis

All statistical analyses were performed using SPSS version 29.0. Statistical significance was set at *P* < 0.05. Data were presented as the mean (±standard deviation) or median (interquartile range) for quantitative measures and as the number of participants (percentage) for qualitative measures. Baseline differences in variables among those with and without new‐onset sarcopenia were analyzed using the *χ* ^2^ test or Fisher's exact test for categorical variables and using the unpaired *t*‐test or Mann–Whitney *U* test for continuous variables.

To examine the association between prescribed medications and new‐onset frailty and sarcopenia, hazard ratios (HRs) and 95% confidence intervals (CIs) were calculated using bivariate and multivariable models, adjusting for age, BMI, cognitive function, depressive symptoms, and daily food diversity score. Ratios were also adjusted for baseline status of sex, educational level, annual income, living arrangement, exercising habit, current alcohol use, individual chronic conditions (i.e., hypertension, diabetes mellitus, dyslipidemia, osteoporosis, malignant neoplasm, stroke, chronic renal failure, and heart disease), and medications (polypharmacy and PIMs use). Multiple imputations using fully conditional specification (chained equations) were applied to impute the missing values for covariates, and 10 datasets were created.

## Results

### Study participants

Of the 2044 participants who completed the baseline assessment, 1549 (mean age, 72.5 ± 5.5 years; 49.1% women) were eligible for inclusion. A total of 216 were excluded owing to baseline sarcopenia (*n* = 168) or the presence of pacemakers (*n* = 6), or missing data (*n* = 4). During the 9‐year follow‐up, 317 participants were lost to follow‐up. Over the 9‐year period (median follow‐up: 6.0 years [interquartile range: 4.0–9.0 years]), 230 participants (14.8%) developed sarcopenia. Additionally, 130 individuals with baseline frailty were excluded from the frailty analysis. Among the remaining 1419 participants, 274 (19.3%) developed frailty. A diagram outlining the study methodology is presented in Figure S1.

### Factors associated with new‐onset sarcopenia

Table 1 presents the baseline characteristics of participants stratified by new‐onset sarcopenia. Older age, physical weakness (low handgrip strength and slower gait speed), psychological deterioration (reduced cognitive function and presence of depressive symptoms), and low serum albumin and hemoglobin levels were more prevalent among those who developed sarcopenia. Minimal differences were observed in gait speed, cognitive function, depressive symptoms, and blood test results (serum albumin, total cholesterol, and hemoglobin levels). Participants with hypertension, heart disease, malignant neoplasms, and comorbidities had a higher likelihood of developing sarcopenia. Additionally, the factors of living alone, low income, lack of exercise, alcohol consumption, dyslipidemia, and chronic renal failure were associated with the likelihood of developing sarcopenia, but this association did not reach statistical significance (*P* < 0.150). The number of medications, polypharmacy, PIM use, and anticholinergic drug use were significantly higher among those who developed sarcopenia. No differences were observed in factors associated with frailty development between the groups.

**Table 1:** Baseline characteristics of participants

Baseline conditions	Overall	Sarcopenia development^†^		*P* ^‡^
		No onset	New onset	
Number of individuals	1549	1319	230	
Basic attributes and daily behavior				
Age, years	72.5 ± 5.5	72.0 ± 5.0	75.4 ± 5.6	<0.001
Sex, women	764 (49.3)	655 (49.7)	109 (47.4)	0.54
Education, ≥ college degree	617 (39.8)	527 (40.0)	90 (39.0)	0.88
Living arrangement, alone	170 (11.0)	137 (10.4)	33 (14.3)	0.08
Low yearly income	311 (20.1)	254 (19.3)	57 (24.7)	0.05
Exercise habit	1257 (81.1)	1081 (82.0)	176 (76.2)	0.06
Food diversity score	4.0 (2.0–5.0)	4.0 (2.0–5.0)	4.0 (2.0–5.0)	0.55
Alcohol habit, daily	769 (49.6)	665 (50.4)	104 (45.0)	0.15
Psychological status				
MMSE score	28.3 ± 1.8	28.4 ± 1.7	27.8 ± 2.0	<0.001
GDS‐15 score	2.0 (0.0–4.0)	1.0 (0.0–4.0)	2.0 (1.0–5.0)	<0.001
Physical status (men/women)				
Body mass index, kg/m^2^	23.4 ± 2.7/22.6 ± 3.2	23.7 ± 2.7/22.8 ± 3.2	22.2 ± 2.6/21.2 ± 2.9	<0.001
Appendicular SMI, kg/m^2^	7.37 ± 0.6/5.92 ± 0.6	7.46 ± 0.6/6.01 ± 0.6	6.84 ± 0.7/5.40 ± 0.4	<0.001
Handgrip strength, kg	35.6 ± 5.4/23.0 ± 3.5	36.3 ± 5.3/23.4 ± 3.4	31.5 ± 4.1/20.5 ± 2.7	<0.001
Usual gait speed, m/s	1.49 ± 0.2/1.49 ± 0.2	1.51 ± 0.2/1.50 ± 0.2	1.40 ± 0.2/1.39 ± 0.2	<0.001
Biochemical parameters				
Serum albumin, g/dL	4.43 ± 0.22	4.44 ± 0.22	4.40 ± 0.22	0.007
Total cholesterol, mg/dL	213 ± 33	213 ± 33	208 ± 34	0.05
Hemoglobin, g/dL	14.0 ± 1.3	14.0 ± 1.3	13.7 ± 1.2	<0.001
C‐reactive protein, mg/dL	0.12 ± 0.3	0.11 ± 0.3	0.14 ± 0.4	0.19
Platelet count per 10^4^ μL	21.8 ± 5.8	21.8 ± 5.9	21.6 ± 5.4	0.66
Chronic conditions				
Hypertension	669 (43.2)	552 (41.8)	117 (50.6)	0.011
Dyslipidemia	620 (40.0)	518 (39.3)	102 (44.2)	0.15
Heart disease	261 (16.8)	206 (15.6)	55 (23.8)	0.002
Malignant neoplasm	230 (14.8)	183 (13.9)	47 (20.3)	0.010
Diabetes mellitus	192 (12.4)	161 (12.2)	31 (13.4)	0.59
Osteoporosis	149 (9.6)	122 (9.2)	27 (11.7)	0.24
Stroke	94 (6.1)	76 (5.8)	18 (7.8)	0.22
Chronic renal failure	12 (0.8)	8 (0.6)	4 (1.7)	0.09
Comorbidity, ≥2 diseases	664 (42.9)	539 (40.9)	125 (54.3)	<0.001
Prescribed medications				
Number of medications	2.0 (0.0–4.0)	1.0 (0.0–4.0)	2.0 (1.0–5.0)	<0.001
Polypharmacy, ≥ 5 drugs	367 (23.6)	273 (20.6)	94 (40.7)	<0.001
PIMs use	436 (28.0)	346 (26.1)	90 (39.0)	<0.001
Anticholinergic drugs use	331 (21.3)	258 (19.5)	73 (31.6)	<0.001

Data are presented as the mean (±standard deviation) or median (interquartile range) for quantitative measures and as the number of participants (percentages) for all qualitative measures.

GDS‐15, Geriatric Depression Scale‐15; MMSE, Mini‐Mental State Examination; PIM, potentially inappropriate medication; SMI, skeletal muscle mass index.

^†^ 130 individuals with frailty at the baseline survey were excluded from the analysis of frailty outcome.

^‡^ Baseline differences in variables among those with/without new‐onset sarcopenia were analyzed using the *χ* ^2^ test or Fisher's exact test for categorical variables and the unpaired *t*‐test or Mann–Whitney *U* test for continuous variables.

### Prevalence and impact of prescribed medications and anticholinergic burden

**Figure 1:**
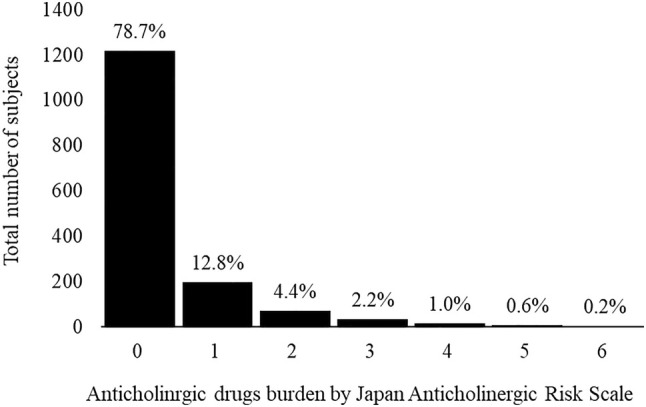
Histogram on the Japan Anticholinergic Risk Scale score at baseline.

The median number of prescribed medications was 2.0 (interquartile range: 0–4; range: 0–17), with 428 individuals (27.6%) having no prescribed medications. A total of 367 individuals were prescribed more than five drugs (i.e., polypharmacy), and, according to the STOPP‐J criteria,^13^ 436 individuals (28.1%) were prescribed PIMs. Furthermore, 331 individuals (21.3%) were taking medications listed on the Anticholinergic Risk Scale developed by the Japanese Society of Geriatric Pharmacology.^14^ Each drug and the number of prescriptions are summarized in Table 2. Of the 158 drugs listed on the Japanese Anticholinergic Drug Risk Scale, 75 were prescribed. The total anticholinergic load was calculated using this scale, and the histogram is presented in Figure 1. The distribution of ACB scores was as follows: 12.8% for score 1, 4.4% for score 2, and >4.0% for score 3. The maximum ACB score recorded was 6.

**Table 2:** Total number of participants receiving each medication listed in the Japan Anticholinergic Risk Scale score at baseline

Medications (score)	*n*	Medications (score)	*n*	Medications (score)	*n*
Triazolam (1)	24	Difenidol (3)	1	Dicycloverine/dicyclomine (3)	1
Estazolam (1)	1	Digoxin (1)	14	Trimebutine (1)	5
Flunitrazepam (1)	4	Isosorbide mononitrate (1)	13	Domperidone (1)	5
Alprazolam (1)	11	Isosorbide dinitrate (1)	3	Dexamethasone (1)	1
Chlordiazepoxide (1)	1	Dipyridamole (1)	4	Prednisolone (1)	14
Lorazepam (1)	3	Disopyramide (2)	5	Imidafenacin (3)	6
Diazepam (1)	5	Amiodarone (1)	3	Solifenacin (3)	13
Clonazepam (1)	4	Captopril (1)	2	Tolterodine (3)	2
Phenobarbital (1)	5	Trandolapril (1)	1	Oxybutynin (3)	1
Carbamazepine (2)	3	Diltiazem (1)	16	Propiverine (3)	4
Valproic acid (1)	8	Nifedipine (1)	59	Flavoxate (3)	1
Carbidopa ± levodopa (1)	8	Atenolol (1)	26	Warfarin (1)	43
Pramipexole (1)	3	Metoprolol (1)	5	Colchicine (1)	1
Trihexyphenidyl/Benzhexol (3)	2	Hydralazine (1)	1	Metformin (1)	8
Biperiden (3)	2	Furosemide (1)	31	Methotrexate (1)	7
Entacapone (1)	2	Cloperastine (2)	1	Chlorpheniramine (3)	9
Chlorpromazine (3)	2	Dextromethorphan (1)	5	Hydroxyzine (3)	1
Fluphenazine (2)	1	Codeine (1)	3	Mequitazine (3)	4
Haloperidol (1)	1	Theophylline (2)	7	Cetirizine (2)	21
Lithium (1)	1	Tiquizium (3)	1	Epinastine (1)	3
Amitriptyline (3)	2	Loperamide (1)	1	Olopatadine (1)	17
Setiptiline (2)	1	Cimetidine (2)	5	Fexofenadine (1)	14
Maprotiline (2)	2	Nizatidine (1)	13	Loratadine (1)	6
Mirtazapine (1)	2	Famotidine (1)	31	Celecoxib (1)	25
Eperisone (2)	13	Lansoprazole (1)	81	Tramadol (2)	1

Among 158 drugs listed in the Japanese Anticholinergic Drug Risk Scale, 75 drugs were prescribed. Eighty‐three drugs that were not prescribed to the participants in this study are not listed in this table.

### Anticholinergic drug burden as a predictor of new‐onset frailty and sarcopenia

**Figure 2:**
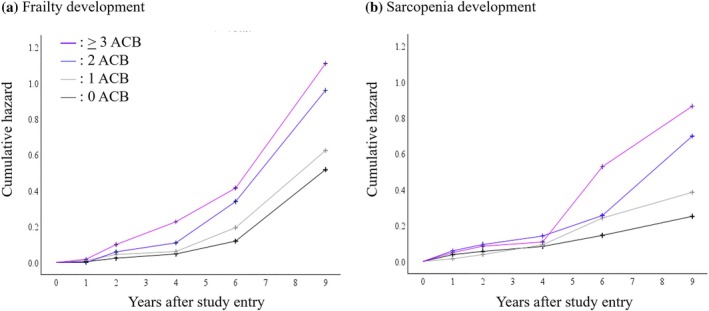
Cumulative hazard curves for (a) frailty development and (b) sarcopenia development with a 9‐year follow‐up according to the risk score of anticholinergic drugs. ACB, anticholinergic drug burden. The results of survival analysis according to the Kaplan–Meier curves showed a log‐rank (Mantel–Cox) of 30.3 (3), *P* < 0.001, for frailty, and a log‐rank (Mantel–Cox) of 28.8 (3), *P* < 0.001, for sarcopenia.

We examined the association between anticholinergic drug burden, as determined using the Japanese Anticholinergic Drug Risk Scale, and new‐onset frailty and sarcopenia over the 9‐year follow‐up. Figure 2 presents the Kaplan–Meier hazard curves for new‐onset frailty and sarcopenia by the ACB group. The survival analysis showed a log‐rank (Mantel–Cox) of 30.3 (*P* < 0.001) for frailty and of 28.8 (*P* < 0.001) for sarcopenia. Specifically, we observed no significant difference in sarcopenia incidence during the first 4 years of follow‐up, but an improvement in the cumulative hazard ratio was observed at 4–6 years.

The covariate‐adjusted HRs and 95% CIs for frailty and sarcopenia development based on ACB are presented in Table 3. After adjusting for covariates, older adults with an ACB score of 2 and those with an ACB score of >3 showed an increased frequency of new‐onset frailty and sarcopenia. In models adjusted for potential confounders, including polypharmacy and PIM use, a significant increase in HRs was observed only in the group with ACB scores of >3 (frailty development: 41.8% vs. 17.4%; sarcopenia development: 35.5% vs. 12.9%).

**Table 3:** Longitudinal association of anticholinergic drugs burden using the Japan Anticholinergic Risk Scale with new‐onset sarcopenia and frailty

Japan Anticholinergic Risk Scale score	Sarcopenia development				Frailty development			
	*N*	Number of cases (%)	Unadjusted HR (95% CI)	Adjusted HR (95% CI)^†^	*N*	Number of cases (%)	Unadjusted HR (95% CI)	Adjusted HR (95% CI)^‡^
No. of individuals	1419	274 (19.3)			1549	230 (14.8)		
Continuous								
Per +1 ACB	‐	‐	**1.35 (1.21–1.49)**	**1.31 (1.16–1.49)**	‐	‐	**1.34 (1.21–1.48)**	**1.24 (1.09–1.40)**
Categorical								
0 ACB	1131	197 (17.4)	1.00 (reference)	1.00 (reference)	1220	157 (12.9)	1.00 (reference)	1.00 (reference)
1 ACB	175	36 (20.6)	1.31 (0.92–1.87)	1.27 (0.87–1.87)	198	34 (17.2)	1.38 (0.95–2.00)	1.24 (0.83–1.84)
2 ACB	58	18 (31.0)	**2.01 (1.24–3.26)**	1.26 (0.72–2.20)	69	17 (24.6)	**2.02 (1.23–3.33)**	1.36 (0.76–2.41)
≥3 ACB	55	23 (41.8)	**2.47 (1.60–3.81)**	**2.45 (1.52–3.94)**	62	22 (35.5)	**2.73 (1.75–4.27)**	**2.01 (1.20–3.35)**

HRs and 95% CI were calculated using the Cox proportional hazards model. Bold typeface indicates statistical significance (*P* < 0.05).

ACB, anticholinergic burden; CI, confidence interval; HR, hazard ratio.

^†^ A total of 130 individuals with frailty at the baseline survey were excluded from the analysis.

^‡^ The multivariate model included the following potentially confounding baseline factors: age, sex, education level (college degree or less), low annual income, body mass index, living alone, cognitive function, depressive symptoms, exercise habits, daily food diversity, alcohol habits, chronic diseases (hypertension, diabetes mellitus, dyslipidemia, osteoporosis, malignant neoplasm, stroke, chronic renal failure, heart disease), and medications (polypharmacy and potentially inappropriate medications use).

## Discussion

In this study, we examined the long‐term relationship between anticholinergic drug burden and the risk of frailty and sarcopenia in community‐dwelling older adults. Our findings indicate that a higher anticholinergic burden, assessed using the newly developed Japanese Anticholinergic Drug Risk Scale, was associated with an increased risk of frailty and sarcopenia over a 9‐year follow‐up period. We observed that older adults with an anticholinergic burden score of ≥3 had a significantly increased risk of developing frailty and sarcopenia. For sarcopenia, the cumulative hazard rate improved between 4 and 6 years after the start of the follow‐up. This suggests that long‐term use of anticholinergic drugs may gradually reduce muscle function, physical resilience, and cognitive function, contributing to sarcopenia and frailty.

Anticholinergic drugs block muscarinic receptors of the neurotransmitter acetylcholine, which plays a crucial role in the cholinergic nervous system. Muscarinic receptors (subtypes M1–M5) are widely distributed in the peripheral nervous system, including in the organs of the autonomic nervous system as well as in those of the central nervous system, such as the brain and spinal cord.^21^ Multiple drugs with anticholinergic effects, when administered simultaneously, additively block muscarinic receptors, leading to adverse events. Even when the anticholinergic effect of individual drugs is minimal, their combined use can lead to an increased “anticholinergic burden.”^22^, ^23^ This can result in side effects, such as cognitive decline, urinary incontinence, dry mouth, and gastrointestinal disturbances.^8^ Anticholinergic drugs may also impair neuromuscular transmission, reduce muscle strength, and decrease physical function, contributing to the development of sarcopenia and frailty. Many studies have reported an association between anticholinergic drug use and negative outcomes such as falls and poor physical function, as assessed using anticholinergic risk scales developed outside of Japan.^9^, ^10^, ^11^, ^12^, ^13^

Additionally, anticholinergic drug use has diverse side effects, including decreased appetite and oral function, and is associated with dry mouth, tooth decay, and swallowing disorders.^24^, ^25^, ^26^ Oral frailty, a decline in oral function in older adults, is also a predictor of future sarcopenia, frailty, and mild cognitive impairment.^27^, ^28^ Thus, side effects of anticholinergic drug on oral function and food intake may lead to sarcopenia and frailty.

Our findings support the need to comprehensively evaluate the anticholinergic burden, especially in older adults with polypharmacy. Polypharmacy is particularly common among older adults with frailty tendencies and high care levels.^29^, ^30^ Use of the Japanese Anticholinergic Drug Risk Scale in community healthcare settings and pharmacies can help identify high‐risk individuals and implement appropriate interventions crucial for preventing frailty and sarcopenia. Furthermore, the Japanese Anticholinergic Drug Risk Scale has no age restrictions. Because the carry‐over effect of sarcopenia is expected at a younger age, appropriate measures should be taken regardless of the generation.^31^ Community healthcare providers should incorporate regular evaluations of anticholinergic burden into clinical practice, especially for older adults with multiple diseases and polypharmacy. This can be achieved using evaluation tools such as the STOPP‐J and the Japanese Anticholinergic Drug Risk Scale.^8^, ^16^ However, a meta‐analysis of intervention trials aimed at deprescribing anticholinergic drugs did not significantly reduce anticholinergic scores,^32^ mainly because of short follow‐up periods and a lack of training and support for successful deprescribing. Therefore, training and educational programs for healthcare professionals on the impact of anticholinergic burden and the use of anticholinergic risk scales may enhance informed decision‐making in medication management for older adults. Policymakers should also raise awareness of the risks associated with anticholinergic drugs and promote public health measures that encourage safe prescription practices, improving health outcomes in older adults.

We employed a robust longitudinal design with a 9‐year follow‐up period, which facilitated the assessment of the long‐term effects of anticholinergic burden on frailty and sarcopenia. However, the study population was limited to community‐dwelling older adults in Kashiwa, Japan, which may limit the generalizability of our findings to other populations. Additionally, residual confounding owing to changes in medications and lifestyle habits during the follow‐up period cannot be entirely ruled out. Further, this scale does not adjust scores by dosage owing to the quantitative nature of the ACB score. The influence of different doses on the results cannot be ruled out.^8^

## Conclusion

In conclusion, our findings suggest that the anticholinergic burden, evaluated using the Japanese Anticholinergic Drug Risk Scale, was a predictor of frailty and sarcopenia in community‐dwelling older adults. These findings highlight the importance of comprehensive evaluation and management of anticholinergic burden to promote healthy aging and prevent adverse health outcomes. Future research must be conducted to validate these findings in diverse populations and explore targeted interventions to mitigate the impact of the anticholinergic burden in older adults.

## Funding information

This study was supported by a Health and Labor Sciences Research Grant (grant no. H24‐Choju‐Ippan‐002) provided by the Ministry of Health, Labor, and Welfare of Japan; the Japan Agency for Medical Research and Development under grant number JP21km0908001; and the Institute for Health Economics and Policy, Japan. The financial sponsor played no role in the design, methods, individual recruitment, data collection, analysis, or preparation of this study.

## Disclosure statement

The authors declare no conflict of interest.

## Author contributions

Study concept and design: Tomoki Tanaka, Masahiro Akishita, and Katsuya Iijima. Acquisition of participants: Tomoki Tanaka, Masahiro Akishita, and Katsuya Iijima. Data analysis and interpretation: Tomoki Tanaka, Masahiro Akishita, Taro Kojima, Bo‐Kyung Son, and Katsuya Iijima. Manuscript preparation: Tomoki Tanaka, Masahiro Akishita, Taro Kojima, Bo‐Kyung Son, and Katsuya Iijima. The corresponding author (Katsuya Iijima) attests that all listed authors meet the authorship criteria and that no others meeting the criteria have been omitted. All the authors affirm that the manuscript is an honest, accurate, and transparent account of the study being reported, that no important aspects of the study have been omitted, and that any discrepancies from the study, as originally planned, have been explained.

## Ethics statement

The study protocol was approved by the Ethics Committee of the University of Tokyo Life Science Research Center (21‐192).

## Patient consent

Written informed consent was obtained from all the participants. The study was conducted in accordance with the principles of the Declaration of Helsinki.

## Supporting information


**Data S1.** Supporting Information.

## Data Availability

The data that support the findings of this study are available from the corresponding author upon reasonable request.
