# Supplementary figures and images for: Anticholinergic burden quantified using the Japanese risk scale as a predictor of frailty and sarcopenia among community‐dwelling older adults: A 9‐year Kashiwa cohort study
Source: Geriatr Gerontol Int. 2025 Mar 6;25(4):520–7. doi: 10.1111/ggi.70012 (PMC11973022; doi:10.1111/ggi.70012)

**
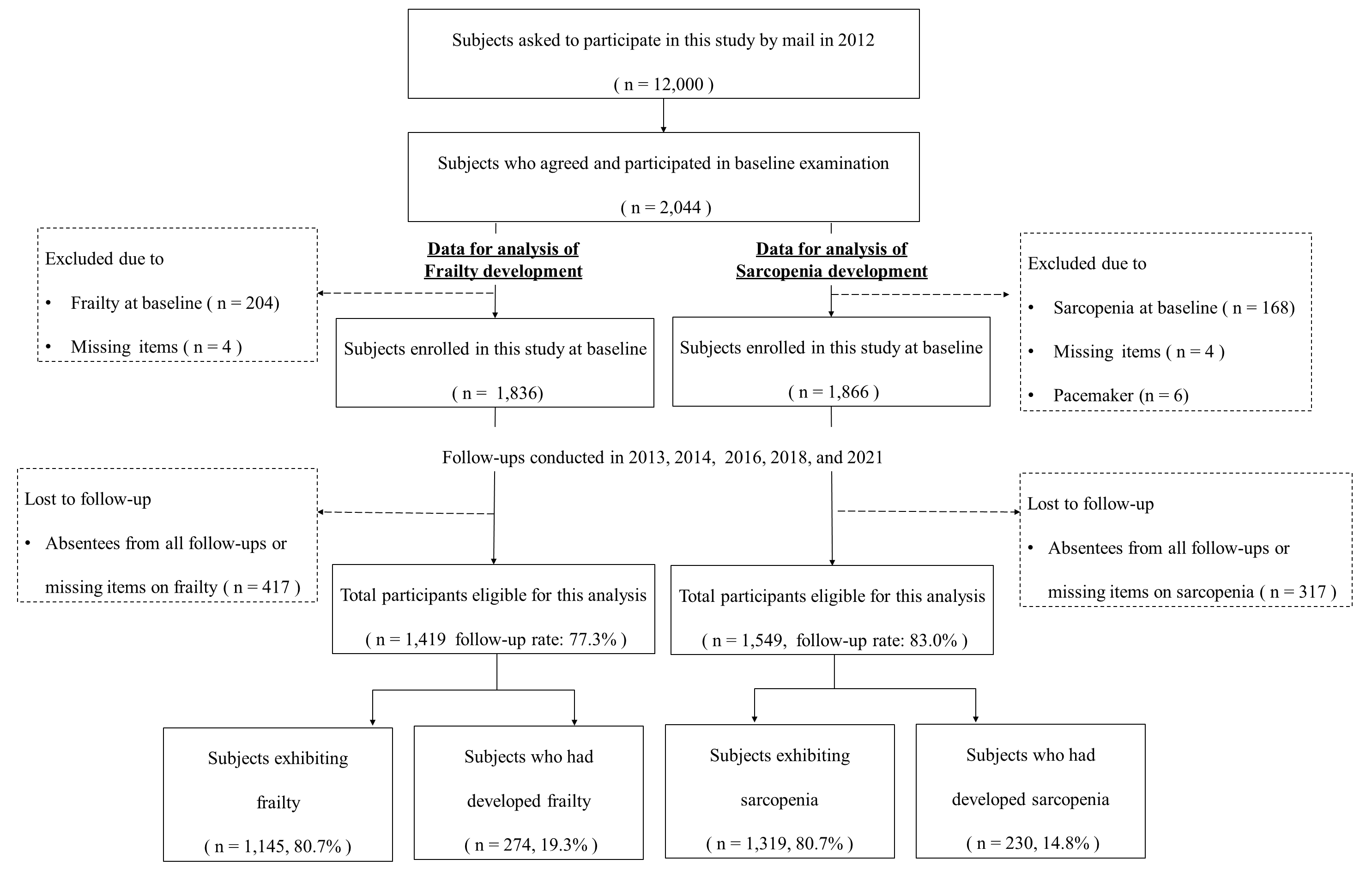
**

**Supplementary Figure S1.** Study diagram

Supplement: Supplementary file 1 — Data S1. Supporting Information. [file GGI-25-520-s001.docx]
